# *CYP2A6* Polymorphisms Associate with Outcomes of S-1 Plus Oxaliplatin Chemotherapy in Chinese Gastric Cancer Patients

**DOI:** 10.1016/j.gpb.2016.11.004

**Published:** 2017-08-12

**Authors:** Lin Yang, Shanshan Zou, Chang Shu, Yan Song, Yong-Kun Sun, Wen Zhang, Aiping Zhou, Xinghua Yuan, Yi Yang, Songnian Hu

**Affiliations:** 1 Department of Medical Oncology, National Cancer Center/Cancer Hospital, Chinese Academy of Medical Sciences and Peking Union Medical College, Beijing 100021, China; 2 CAS Key Laboratory of Genome Sciences and Information, Beijing Institute of Genomics, Chinese Academy of Sciences, Beijing 100101, China; 3 Department of Pancreatic and Gastric Surgery, National Cancer Center/Cancer Hospital, Chinese Academy of Medical Sciences and Peking Union Medical College, Beijing 100021, China

**Keywords:** *CYP2A6*, Gastric cancer, Pharmacogenomics, Polymorphism, SOX

## Abstract

Gastric carcinoma is a heterogeneous malignant disease involving genetic factors. To identify predictive markers for **gastric cancer** treatment in Chinese patients, we evaluated the association between **polymorphisms** of the gene encoding cytochrome P450 2A6 (***CYP2A6***) and outcomes of S-1 plus oxaliplatin (**SOX**) chemotherapy treatment. Clinical data on 60 consecutive gastric cancer patients receiving SOX regimen were collected prospectively. We sequenced all exons of *CYP2A6* and a total of 22 different polymorphisms were detected in the present study. Comprehensive analyses of these genetic polymorphisms were performed to determine their association with both safety and efficacy of SOX regimen. Our results showed that polymorphisms of *CYP2A6* were associated with the safety and efficacy of SOX treatment. Among them, missense mutations *CYP2A6* rs60823196 and rs138978736 could be possible risk factors (*P* < 0.05) for severe diarrhea induced by SOX, whereas *CYP2A6* rs138978736 could be a conceivable predictor for overall survival of patients treated with SOX adjuvant chemotherapy. Further large-scale randomized prospective studies are warranted to confirm these findings.

## Introduction

Gastric cancer is a leading cause of cancer-related death all over the world with around 50% of gastric carcinoma patients from East Asia [1], [2]. In China, gastric cancer each year accounts for about 35%–40% of new cases and deaths globally [1], with its national incidence and mortality both in third place [3].

Surgical removal remains the cornerstone for resectable gastric carcinoma. However, the high rate of recurrence and metastasis, as well as the poor survival following resection, makes it necessary to consider postoperative adjuvant treatment [4], [5]. As a first-line regimen for gastric cancer patients, the efficacy and safety of S-1 plus oxaliplatin (SOX) combination chemotherapy have been extensively validated [6], [7], [8]. Notably, a good response rate has been achieved in patients suffering from resectable advanced gastric adenocarcinoma, upon SOX treatment after surgery [5].

S-1 is an oral fluoropyrimidine [9], [10] used as an adjuvant treatment agent for stomach carcinoma [11], [12]. The main antitumor ingredient of S-1 is tegafur, which is converted to 5-fluorouracil (5-FU) catalyzed primarily by cytochrome P450 2A6 (CYP2A6) [13], [14]. CYP2A6 exhibits varied enzyme activity (http://www.cypalleles.ki.se) and polymorphic variations in *CYP2A6* are detected more frequently in Asians than in Caucasians [15], [16], [17]. *CYP2A6* has been reported to be associated with clinical outcomes of S-1-based regimen for patients with gastric carcinoma [18], [19]. Recent pharmacokinetic studies have also revealed that gastric cancer patients treated with S-1 possess different plasma concentrations and clearance of tegafur, due to the *CYP2A6* polymorphisms [20].

Oxaliplatin is a platinum analog showing strong inhibitory effect on DNA synthesis and well-tolerated adverse reactions [21], [22]. Oxaliplatin targets DNA and forms Pt-DNA adducts, thus leading to blockage of DNA replication and death of tumor cells [23].

In the present study, we set out to identify genetic variations by Sanger sequencing to evaluate correlations between genetic variants of *CYP2A6* and outcomes of gastric carcinoma patients treated with SOX chemotherapy to identify potential markers to assist in therapeutic selection.

## Results

### Patient characteristics and treatment outcomes

A total of 60 eligible patients were included in the present study. These included 30 patients who were treated with SOX as first-line chemotherapy and the other 30 patients who were treated with SOX as adjuvant chemotherapy after surgery. Basic characteristics, survival information, and severe hematological and non-hematological toxicity of SOX regimens are summarized in Table 1. These two groups were comparable in ECOG performance status but varied a lot in survival time. Toxicity was found in 3.3%−26.7% of all patients in terms of the occurrence of severe neutropenia, thrombocytopenia, diarrhea, vomiting, and nausea. More patients receiving SOX as the first-line treatment appeared to suffer from severe neutropenia, thrombocytopenia and diarrhea than those receiving SOX as the adjuvant therapy, while the appearance of vomiting and nausea was comparable in both groups.

**Table 1:** **Demographic and clinical features of 60 gastric cancer patients examined in the current study**

**Feature**		**No. (percentage) of patients**		
		**All (*n* = 60)**	**SOX only (*n* = 30)**	**Surgery + SOX (*n* = 30)**
Median age (range)		54 (27–75)	55 (36–75)	52 (27–70)

Gender	Male	46 (76.7%)	20 (66.7%)	26 (86.7%)
	Female	14 (23.3%)	10 (33.3%)	4 (13.3%)

ECOG performance status	0	22 (36.7%)	9 (30.0%)	13 (43.4%)
	1	34 (56.7%)	18 (60.0%)	16 (53.3%)
	2	4 (6.7%)	3 (10.0%)	1 (3.3%)

RECIST	CR + PR	15 (25.0%)	15 (50.0%)	—
	SD	4 (6.7%)	4 (13.3%)	—
	PD	9 (15.0%)	9 (30.0%)	—
	NE	32 (53.3%)	2 (6.7%)	—

Median OS (months, range)		—	12.0 (3.5–52.0)	42.5 (22.0–50.0)

Median PFS (months, range)		—	6.0 (1.5–52.0)	41.5 (3.0–50.0)

Neutropenia grade 3–4		16 (26.7%)	9 (30.0%)	6 (20.0%)

Thrombocytopenia grade 3–4		12 (20.0%)	7 (23.3%)	4 (13.3%)

Diarrhea grade 3–4		6 (10.0%)	5 (16.7%)	1 (3.3%)

Vomiting grade 3–4		5 (8.3%)	2 (6.7%)	3 (10.0%)

Nausea grade 3–4		2 (3.3%)	1 (3.3%)	1 (3.3%)

*Note*: ECOG, Eastern Cooperative Oncology Group; RECIST, Response Evaluation Criteria In Solid Tumors; CR, complete response; PR, partial response; SD, stable disease; PD, progressive disease; NE, not evaluable; OS, overall survival; PFS, progression-free survival.

### Association between *CYP2A6* polymorphisms and severe toxicity

To examine the possible effects of CYP2A6 on the drug toxicity of SOX therapy, we sequenced all exons of *CYP2A6* for single nucleotide polymorphisms (SNPs) present in the 60 patients. As a result, we identified totally 22 SNPs in the present study, all of which were in Hardy–Weinberg equilibrium (*P* > 0.01). The allele designations were defined according to the CYP Allele Nomenclature Committee and the identified polymorphisms in *CYP2A6*are listed in Table S1.

CYP2A6*5,*7,*8,*10, and *11 were found with occurrence ranging 0.031–0.062 (Table S1). The allelic frequencies of CYP2A6*5,*7,*8, and *10 were similar to those reported previously [18], [19], [24], whereas frequency of CYP2A6*11, which showed poor metabolic phenotype toward tegafur, has not been definitely determined before [25]. Previous studies have shown that CYP2A6*5 and *8 are unlikely to affect catalytic activity, whereas CYP2A6*7,*10, and *11 yield enzyme with reduced activity (http://www.cypalleles.ki.se/cyp2a6.htm). In this study, *CYP2A6* rs5031017 occurring in variant *5 and rs28399468 occurring in variant *8 did not exhibit association with severe toxicity (Table 2), which was consistent with previous reports about their lack of impact on enzyme activity.

**Table 2:** **Correlation between *CYP2A6* polymorphisms and severe diarrhea**

**SNP**	**dbSNP ID**	***P* value**	**OR (CI 95%)**
M01	rs28399468	0.5410	1.422 (0.16–12.82)
M02	rs5031017	1.0000	0
M03	rs5031016	1.0000	0
M04	rs150586234	0.2330	2.450 (0.67–8.93)
M05	rs771265125	0.0750	3.433 (0.92–12.81)
M06	rs779290232	1.0000	0.982 (0.12–8.41)
M07	rs762887319	1.0000	0
M08	rs200267449	0.5010	1.697 (0.19–15.40)
M09	rs58571639	0.2240	2.605 (0.71–9.53)
M10	rs2644907	0.4260	1.737 (0.43–7.01)
M11	rs60988093	0.0890	3.188 (0.86–11.82)
**M12**	**rs60823196**	**0.0200**	**4.905 (1.38–17.45)**
M13	rs4997557	0.2330	2.450 (0.67–8.93)
M14	rs2644906	0.5010	1.697 (0.19–15.40)
M15	rs2644905	0.0890	3.188 (0.86–11.82)
M16	rs139639589	1.0000	1.101 (0.13–9.52)
M17	rs55805386	1.0000	0
M18	rs140471703	0.0750	3.433 (0.92–12.81)
**M19**	**rs138978736**	**0.0002**	**15.860 (4.05–62.11)**
M20	rs111033610	1.0000	0
M21	rs199515342	1.0000	0.624 (0.075–5.19)
M22	rs200554095	1.0000	0

*Note*: SNP, single nucleotide polymorphism; OR, odds ratio; CI, confidence interval. *P* values were generated using 2-tailed Fisher’s exact test. Significant associations are highlighted in bold.

We then performed association analysis between toxicity phenotypes (shown in Table 1) and *CYP2A6* polymorphisms (shown in Table S1). As shown in Table 2 and Table S2, only SNPs rs60823196 and rs138978736 in *CYP2A6* were significantly associated with grade 3–4 diarrhea (*P* < 0.05; Table 2), whereas no significant associations were found for other SNPs and toxicity phenotypes. The odds ratios are 4.905 with 95% of confidence interval (CI) of 1.38–17.45 for rs60823196 and 15.860 with 95% CI of 4.05–62.11 for rs138978736, respectively, indicating that these two SNPs could be the risk factors for SOX-induced severe diarrhea. Therefore, we focused the following analyses on these two SNPs.

### Association between rs60823196/rs138978736 genotypes and severe diarrhea

We next analyzed the risk of severe diarrhea associated with *CYP2A6* rs60823196 or rs138978736 genotypes (Table 3). SNPs rs60823196 and rs138978736 were found in 30% and 18% of the 60 patients examined in this study. Fisher’s exact test analysis revealed that occurrence of diarrhea varied significantly among patients with wild/heterozygous/homozygous genotypes of *CYP2A6* (*P* = 0.0270 for rs60823196 and *P* < 0.0001for rs138978736). Similarly, significant differences were observed using Cochran-Armitage trend test (*P* = 0.0100 for rs60823196 and *P* < 0.0001 for rs138978736). We therefore speculate that there may be a linear relationship between occurrence of severe diarrhea and variant allele number of *CYP2A6* rs60823196 orrs138978736, *i.e.*, more variant alleles of *CYP2A6* rs60823196 or rs138978736 might lead to higher risk of grade 3–4 diarrhea (Table 3).

**Table 3:** **Correlation analysis and trend test of severe diarrhea and *CYP2A6* rs60823196 or rs138978736 genotypes**

**SNP**	**dbSNP ID**	**Genotype**	**Total**	**Grade 3–4 diarrhea**		**Fisher’s exact test *P* value**	**Cocharan-Armitage trend test *P* value**
				**No**	**Yes**		
M12	rs60823196	Wild type (GG)	42	40	2	0.0270	0.0100
		Heterozygous (GC)	16	13	3		
		Homozygous (CC)	2	1	1		
M19	rs138978736	Wild type (CC)	49	47	2	<0.0001	<0.0001
		Heterozygous (CA)	9	7	2		
		Homozygous (AA)	2	0	2		

Furthermore, patients with homozygous *CYP2A6* rs138978736 variants had significantly higher risk of grade 3–4 diarrhea compared to patients with the wild type genotype (*P* = 0.004), whereas no significant difference was observed for patients when comparing homologous rs60823196 variants with wild-type (Table 4). The risk of severe diarrhea between patients with wild genotype and those carrying one or two variant alleles was also significantly different (*P* < 0.05; Table 4). Patients carrying variant alleles of rs60823196 or rs138978736 had a higher risk of developing severe diarrhea (OR = 6.43, 95%CI = 1.06–38.90 for rs60823196; OR = 14.86, 95%CI = 2.29–96.57 for rs138978736). These data indicated that compared to patients with wild type genotypes, individuals carrying variant alleles were prone to severe diarrhea. We also analyzed the combinatorial effects of these two variants on the risk of severe diarrhea. We found that although there was a significant difference between patients carrying both *CYP2A6* rs60823196 and rs138978736 variant alleles compared to patients with wild type genotypes (*P* = 0.015), patients carrying both *CYP2A6* rs60823196 and rs138978736 variant alleles did not show a higher OR (12.35) than those carrying either rs60823196 (6.43) or rs138978736 variant (14.86) (Table 4).

**Table 4:** **Correlation between severe diarrhea and *CYP2A6* rs60823196 and/or rs138978736 genotypes**

**SNP**	**dbSNP ID**	**Genotype**	**Overall**	**Grade 3**–**4 diarrhea**		***P* value**	**OR (95% CI)**
				**No**	**Yes**		
M12	rs60823196	Wild type (GG)	42	40	2	—	—
		Heterozygous (GC)	16	13	3	0.099	5.19 (0.78–34.46)
		Homozygous (CC)	2	1	1	0.120	22.50 (1.00–505.85)
M19	rs138978736	Wild type (CC)	49	47	2	—	—
		Heterozygous (CA)	9	7	2	0.094	7.43 (0.90–61.44)
		**Homozygous (AA)**	**2**	**0**	**2**	**0.004**	**—**
M12	rs60823196	Wild type (GG)	42	40	2	—	—
		**Others (GC** + **CC)**	**18**	**14**	**4**	**0.045**	**6.43 (1.06–38.90)**
M19	rs138978736	Wild type (CC)	49	47	2	—	—
		**Others (CA** + **AA)**	**11**	**7**	**4**	**0.006**	**14.86 (2.29–96.57)**
M12 + M19	—	Wild type	38	37	1	—	—
		**Others**	**22**	**17**	**5**	**0.015**	**12.35 (1.34–113.71)**

*Note*: *P* values were generated using 2-tailed Fisher’s exact test. OR, odds ratio; CI, confidence interval. Significant correlations are highlighted in bold.

### Association between rs60823196/rs138978736 genotypes and patient survival

**Figure 1:**
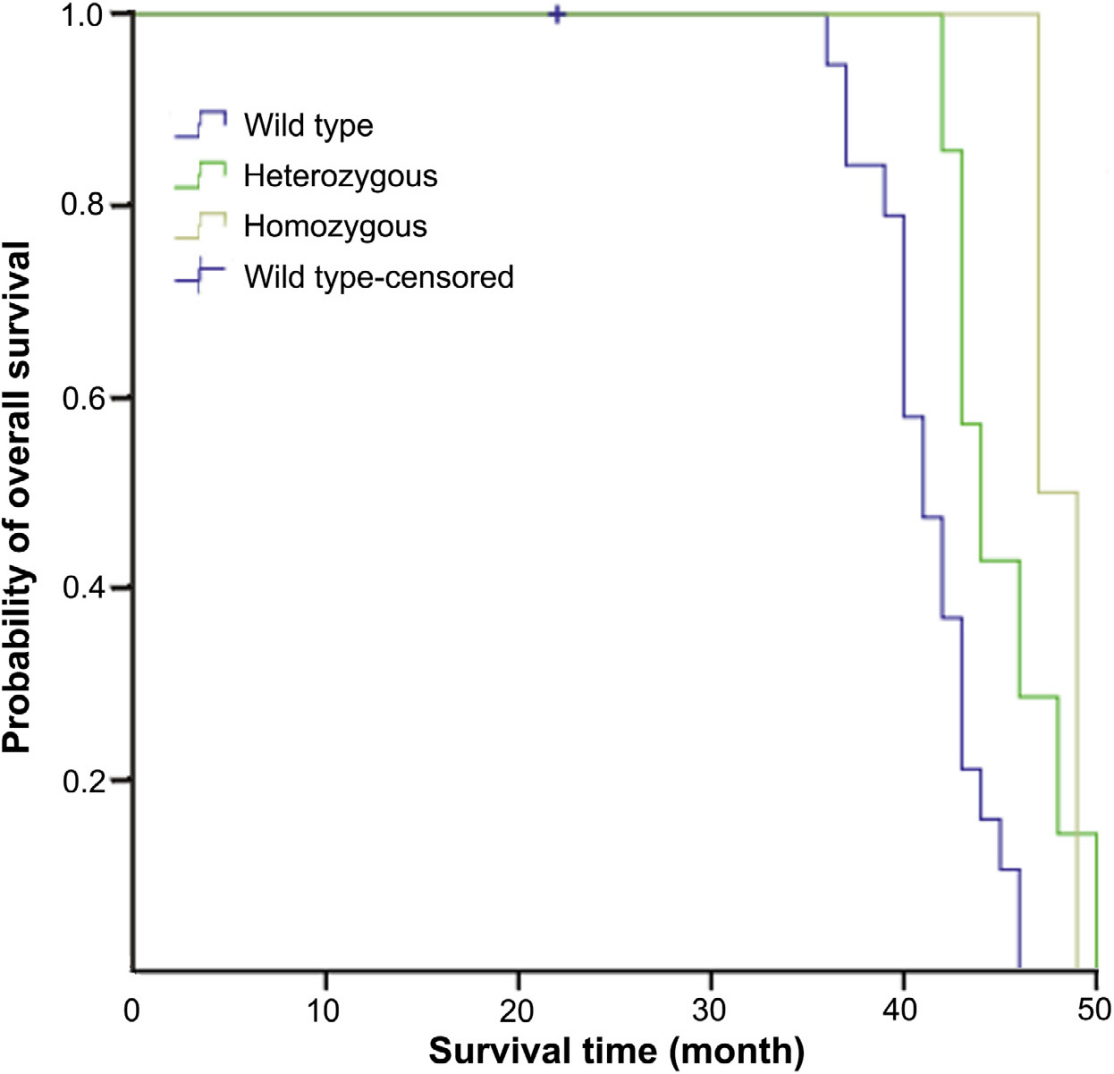
**Kaplan-Meier curve of overall survival stratified based on rs138978736 genotype for gastric cancer patients treated with SOX adjuvant chemotherapy** Log-rank tests were used to perform survival analysis between patients’ genotypes and survival time. The survival curve was plotted according to *CYP2A6* rs138978736 genotypes of 30 gastric cancer patients who were treated with SOX adjuvant chemotherapy (*P* = 0.006). SOX, S-1 plus oxaliplatin.

Association between *CYP2A6* rs60823196/rs138978736 genotypes and survival of patients treated with SOX as first-line and adjuvant regimens was also analyzed. It was shown that *CYP2A6* rs138978736 was a significant independent risk factors for overall survival (OS) for patients treated with SOX as adjuvant chemotherapy (*P* = 0.006) (Figure 1), whereas none of rs60823196 and rs138978736 was significantly associated with OS/progression-free survival (PFS) of patients treated with SOX as first-line chemotherapy.

### Association between *CYP2A6* haplotypes and severe toxicity

**Figure 2:**
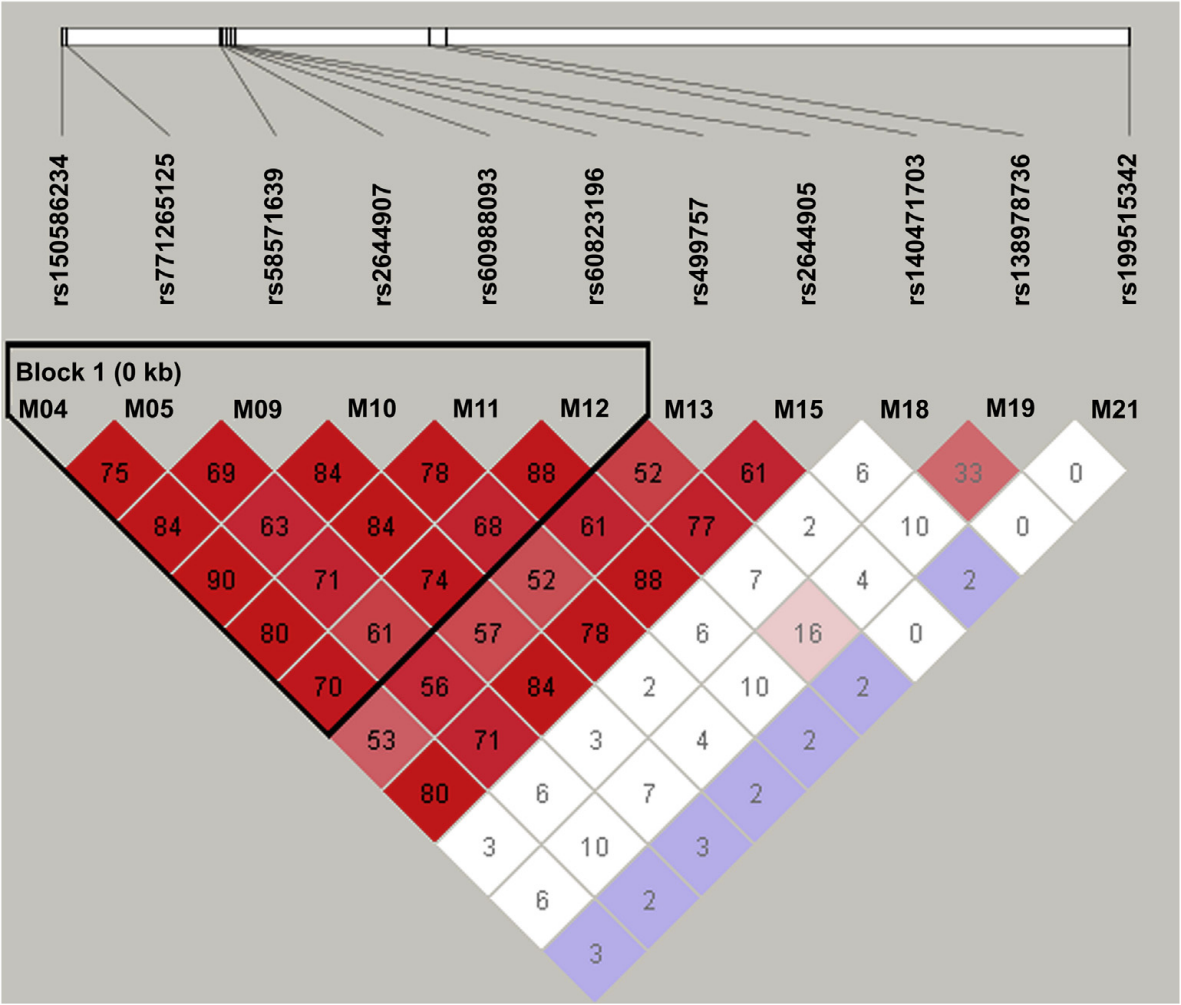
**Pairwise linkage disequilibrium relationships between *CYP2A6* polymorphisms in Chinese gastric cancer patients** The linkage status of variants is displayed in GOLD heatmap color scheme. The thick white line represents the genomic region of CYP2A6 with positions of the identified SNPs indicated in black lines. Diamond with deeper color (from white to red) indicates higher linkage between any two variants. An area corresponding to a haplotype block with *r*^2^ values in the diamonds is boxed (Block 1).

We established haplotypes of SNPs with minimum MAF ≥ 0.1 and estimated its correlation with SOX-induced toxicity (Table 5). Linkage disequilibrium (LD) blocks were defined by the CI algorithm, and LD structure was displayed by Graphical Overview of Linkage Disequilibrium (GOLD) heat map color scheme. As shown in Figure 2, a linkage block was observed across the sequenced region in these Chinese gastric cancer patients.

**Table 5:** **Correlation between *CYP2A6* haplotypes and severe toxicity**

**Haplotype ID**	**Variants covered**						**Frequency**	***P* value**		
	**M04**	**M05**	**M09**	**M10**	**M11**	**M12**		**Neutropenia**	**Diarrhea**	**Vomiting**
H1	C	C	G	G	C	C	0.800	0.413	**0.049**	0.410
H2	T	T	A	C	T	G	0.115	0.659	0.126	0.235
H3	C	C	A	G	T	G	0.015	0.424	**0.046**	0.680
H4	T	C	A	C	C	C	0.015	0.401	0.650	**0.024**
H5	T	C	A	C	T	G	0.015	**0.013**	0.650	0.682

*Note*: *P* values were calculated based on Pearson’s *χ*^2^ test. Significant associations are highlighted in bold.

Five haplotypes were inferred and the most common haplotypes were H1 and H2 which account for 91.5% of all haplotypes (Table 5). Pearson test indicated that haplotypes H1 and H3 were significantly associated with a higher risk of grade 3–4 diarrhea (*P* = 0.049 for H1 and *P* = 0.046 for H3), whereas haplotype H4 showed significant association with grade 3–4 vomiting (*P* = 0.024) and haplotype H5 was significantly associated with grade 3–4 neutropenia (*P* = 0.013).

## Discussion

Polymorphisms in *CYP2A6* are indicated to have association with S-1-based regimen for gastric carcinoma patients repeatedly. Here we conducted direct exon sequencing, aiming to identify variants in *CYP2A6* exon regions that could explain the correlated toxicity and efficacy of SOX regimen for gastric cancer patients.

A total of 22 SNPs from 60 enrolled gastric cancer patients were identified. Despite the lack of significant association between some *CYP2A6* hotspot alleles and severe toxicity in previous studies [19], [24], we found that two missense SNPs identified in the present study, *CYP2A6* rs60823196 and rs138978736, exhibited significant correlation with severe diarrhea. *CYP2A6* rs60823196 and rs138978736 result in amino acid changes at positions 301 (G301A) and 239 (Q239K), respectively. To our best knowledge, there have been no reports about the association between these two SNPs and catalytic activity of CYP2A6 or possible roles of G301 and Q239 in the function of CYP2A6. These SNPs might lead to decreased metabolic activity of S-1 and increased drug toxicity manifested as severe diarrhea described in this study. However, more functional studies and association analyses would be required to test this speculation. In addition, we could not rule out the possibility that variants in non-exonic regions of *CYP2A6*, which were not examined in the current study, might be associated with the severe toxicity of SOX as well [26].

Of these 22 detected variants, variants in CYP2A6*7,*10, and *11 did not exhibit association with severe toxicity. This did not agree with the decreased enzyme activity of these variants (http://www.cypalleles.ki.se/cyp2a6.htm), which might be due to interactions among different variations [27] and regimen heterogeneity [28].

Further association analysis between *CYP2A6* rs60823196 and/or rs138978736 genotypes with clinical features of gastric cancer patients showed that *CYP2A6* rs138978736 was a significant independent risk factor for overall survival of patients treated with SOX as adjuvant chemotherapy. It was worthy of note that patients having fewer *CYP2A6* variants (*4, *7, *9, and *10) had better overall survival when treated with S-1-based chemotherapy [24].

Of the five haplotypes revealed, H1 occurred with highest frequency (80%) and exhibited significant association with severe diarrhea (*P* = 0.049). This could be attributed to the contained missense mutation rs60823196 due to its association with severe diarrhea. However, this association analysis needs to be validated further because the *P* value obtained was close to 0.05. It is also possible that *CYP2A6* rs138978736 is more likely a truly causative variation because *CYP2A6* rs138978736 showed a much lower *P* value (0.0002) in the association test compared with *CYP2A6* rs60823196 (0.0200).

In conclusion, *CYP2A6* rs60823196 and rs138978736 are possible risk factors for serious toxicity in Chinese gastric carcinoma patients treated with SOX chemotherapy. We also show that *CYP2A6* rs138978736 is a significant risk factor for OS of patients treated with SOX as adjuvant chemotherapy. However, given the small sample size in the current study, our findings should be further validated in larger cohorts.

## Materials and methods

### Samples

In total 60 consecutive gastric cancer patients who were treated with SOX chemotherapy were collected in the Cancer Institute and Hospital, Chinese Academy of Medical Sciences (Beijing, China) from 2012 to 2014. Among them, 30 patients who had metastatic disease and measurable lesions were treated with SOX as first-line chemotherapy, while the other 30 patients who had experienced gastrectomy and D2 lymph node resection were treated as adjuvant chemotherapy. All patients met the following eligibility criteria: histologically-confirmed gastric or gastro-esophageal junction adenocarcinoma; Eastern Cooperative Oncology Group (ECOG) performance status of 0–2; age > 18; no coexisting malignancy; adequate organ function; and a sufficient amount of peripheral blood samples for following tests.

All patients were treated with the same SOX regimen. Administrations of S-1 and oxaliplatin were given in our previous studies [28], [29], [30]. All the patients experienced prophylactic anti-emetic medications. Treatment continued until disease progression, unacceptable toxicity, patient refusal, or a medical determination to discontinue treatment. For adjuvant chemotherapy, 8 cycles of chemotherapy were planned. The study protocol was approved by the ethics committee of Cancer Hospital, Chinese Academy of Medical Sciences (Beijing, China), and written informed consent was collected from each patient.

### Assessment of efficacy and adverse event

Patients underwent baseline evaluations as listed in Table 1. Toxicity evaluations were graded according to the National Cancer Institute Common Terminology Criteria for Adverse Events version 4.0. Tumor response was assessed according to Response Evaluation Criteria In Solid Tumors (RECIST) [31]. PFS was calculated from the day that the first chemotherapy cycle started to the day that disease progression was documented or to the date of death from any cause before documented progression, whereas OS was calculated from the day that the first chemotherapy cycle started to the date of death from any cause.

### *CYP2A6* genotyping

Extraction of DNA was performed using the QIAamp DNA Blood Mini Kit (Qiagen, Valencia, CA) and all exons of *CYP2A6* were sequenced to screen the SNPs using the DYEnamic ET Terminator Cycle Sequencing Kit (GE Healthcare, Chalfont St. Giles, UK) on the ABI Prism 3730xl DNA Analyzer (Applied Biosystems, Foster, CA). Primers were designed using Primer 5.0 (Premier Biosoft International, Palo Alto, CA, USA) and the primer sequences are listed in Table S3. PCR reactions were carried out in a final volume of 25 µl, containing 5 ng of genomic DNA, 10× KOD plus buffer (Mg^2+^ plus), 2.5 pM of each primer, 25 pM dNTPs, and 0.5 U of Taq DNA polymerase (KOD plus) (TOYOBO, Shanghai, China). Following pre-denaturation at 94 °C for 3 min, amplification was performed under the following conditions for 35 cycles: denaturation at 94 °C for 45 s; annealing at 58 °C for 40 s; extension at 72 °C for 2 min; and further extension at 72 °C for 10 min.

SNP calling, quality control, and polymorphism confirmation from DNA sequencing were processed using programs developed by University of California, USA (http://elcapitan.ucsd.edu/hyper/polyphred.usage.html).

### Statistical analysis

Correlations between polymorphisms/genotypes and toxicity were analyzed using 2-tailed Fisher’s exact test and were considered significant with 2-sided *P* < 0.05 as calculated by PLINK v1.07 (Shaun Purcell at the Center for Human Genetic Research, Massachusetts General Hospital, and the Broad Institute of Harvard & MIT, Boston, MA). The trend analysis of phenotypes across the genotypes was performed using the Cochran-Armitage trend test in the Statistics Analysis System (SAS), version 9.3 (SAS Institute Inc, Cary, NC).

Kaplan–Meier analysis and the log-rank test were used to conduct univariate analysis between patients’ genotypes and PFS/OS.

Haploview 4.2 (Broad Institute of MIT and Harvard, Cambridge, MA) based on the expectation–maximization method was used to calculate the Lewontin’s coefficients D’ and correlation coefficient *r*^2^, to establish haplotypes and estimate haplotype frequency, and to analyze the relationship between the haplotypes and toxicity. LD blocks were defined by the CI algorithm, and LD structures of SNPs with minimum minor allele frequency ≥0.1 were established by GOLD heatmap color scheme [32], [33].

## Authors’ contributions

SZ, SH, CS, LY, and YY conceived and designed the study. LY, YS, YKS, WZ, AZ, and XY participated in sample processing and data collection. SZ, CS, and SH were involved in data analysis and interpretation. SZ drafted the manuscript. LY provided clinical advice and supervision. SH, LY and SZ revised the manuscript. All authors read and approved the final manuscript.

## Supplementary material

Supplementary Table S1 **Summary of *CYP2A6* SNPs identified in the current study**

Supplementary Table S2 **Correlation between other toxicity phenotypes and *CYP2A6* SNPs**

Supplementary Table S3 **List of *CYP2A6* primers used in the current study**

## References

[1] Jemal A., Bray F., Center M.M., Ferlay J., Ward E., Forman D. (2011). Global cancer statistics. CA Cancer J Clin.

[2] Jung K.W., Won Y.J., Kong H.J., Oh C.M., Seo H.G., Lee J.S. (2013). Cancer statistics in Korea: incidence, mortality, survival and prevalence in 2010. Cancer Res Treat.

[3] Chen W., Zheng R., Zhang S., Zhao P., Zeng H., Zou X. (2014). Report of cancer incidence and mortality in China, 2010. Ann Transl Med.

[4] Alberts S.R., Cervantes A., van de Velde C.J. (2003). Gastric cancer: epidemiology, pathology and treatment. Ann Oncol.

[5] GASTRICG Group, Paoletti X., Oba K., Burzykowski T., Michiels S., Ohashi Y. (2010). Benefit of adjuvant chemotherapy for resectable gastric cancer: a meta-analysis. JAMA.

[6] Kim G.M., Jeung H.C., Rha S.Y., Kim H.S., Jung I., Nam B.H. (2012). A randomized phase II trial of S-1-oxaliplatin versus capecitabine-oxaliplatin in advanced gastric cancer. Eur J Cancer.

[7] Bando H., Yamada Y., Tanabe S., Nishikawa K., Gotoh M., Sugimoto N. (2016). Efficacy and safety of S-1 and oxaliplatin combination therapy i n elderly patients with advanced gastric cancer. Gastric Cancer.

[8] Yamada Y., Higuchi K., Nishikawa K., Gotoh M., Fuse N., Sugimoto N. (2015). Phase III study comparing oxaliplatin plus S-1 with cisplatin plus S-1 in chemotherapy-naive patients with advanced gastric cancer. Ann Oncol.

[9] Fukushima M., Satake H., Uchida J., Shimamoto Y., Kato T., Takechi T. (1988). Preclinical antitumor efficacy of S-1: a new oral formulation of 5-fluorouracil on human tumor xenografts. Int J Oncol.

[10] Shirasaka T., Nakano K., Takechi T., Satake H., Uchida J., Fujioka A. (1996). Antitumor activity of 1 M tegafur-0.4 M 5-chloro-2,4-dihydroxypyridine-1 M potassium oxonate (S-1) against human colon carcinoma orthotopically implanted into nude rats. Cancer Res.

[11] Koizumi W., Narahara H., Hara T., Takagane A., Akiya T., Takagi M. (2008). S-1 plus cisplatin versus S-1 alone for first-line treatment of advanced gastric cancer (SPIRITS trial): a phase III trial. Lancet Oncol.

[12] Sakuramoto S., Sasako M., Yamaguchi T., Kinoshita T., Fujii M., Nashimoto A. (2007). Adjuvant chemotherapy for gastric cancer with S-1, an oral fluoropyrimidine. N Engl J Med.

[13] Ikeda K., Yoshisue K., Matsushima E., Nagayama S., Kobayashi K., Tyson C.A. (2000). Bioactivation of tegafur to 5-fluorouracil is catalyzed by cytochrome P-450 2A6 in human liver microsomes *in vitro*. Clin Cancer Res.

[14] El Sayed Y.M., Sadée W. (1982). Metabolic activation of ftorafur[R,S-1-(tetrahydro-2-furanyl)-5-fluorouracil]: the microsomal oxidative pathway. Biochem Pharmacol.

[15] Mwenifumbo J.C., Myers M.G., Wall T.L., Lin S.K., Sellers E.M., Tyndale R.F. (2005). Ethnic variation in *CYP2A6*7*, *CYP2A6*8* and *CYP2A6*10* as assessed with a novel haplotyping method. Pharmacogenet Genomics.

[16] Kwon J.T., Nakajima M., Chai S., Yom Y.K., Kim H.K., Yamazaki H. (2001). Nicotine metabolism and *CYP2A6* allele frequencies in Koreans. Pharmacogenetics.

[17] Nakajima M., Fukami T., Yamanaka H., Higashi E., Sakai H., Yoshida R. (2006). Comprehensive evaluation of variability in nicotine metabolism and *CYP2A6* polymorphic alleles in four ethnic populations. Clin Pharmacol Ther.

[18] Kim Y.W., Kim M.J., Ryu K.W., Lim H.S., Lee J.H., Kong S.Y. (2015). A phase II study of perioperative S-1 combined with weekly docetaxel in patients with locally advanced gastric carcinoma: clinical outcomes and clinicopathological and pharmacogenetic predictors for survival. Gastric Cancer.

[19] Jeong J.H., Park S.R., Ahn Y., Ryu M.H., Ryoo B.Y., Kong S.Y. (2017). Associations between *CYP2A6* polymorphisms and outcomes of adjuvant S-1 chemotherapy in patients with curatively resected gastric cancer. Gastric Cancer.

[20] Fujita K., Yamamoto W., Endo S., Endo H., Nagashima F., Ichikawa W. (2008). *CYP2A6* and the plasma level of 5-chloro-2,4-dihydroxypyridine are determinants of the pharmacokinetic variability of tegafur and 5-fluorouracil, respectively, in Japanese patients with cancer given S-1. Cancer Sci.

[21] Di Francesco A.M., Ruggiero A., Riccardi R. (2002). Cellular and molecular aspects of drugs of the future: oxaliplatin. Cell Mol Life Sci.

[22] Eriguchi M., Nonaka Y., Yanagie H., Yoshizaki I., Takeda Y., Sekiguchi M. (2003). A molecular biological study of anti-tumor mechanisms of an anti-cancer agent oxaliplatin against established human gastric cancer cell lines. Biomed Pharmacother.

[23] Hildebrandt M.A., Gu J., Wu X. (2009). Pharmacogenomics of platinum-based chemotherapy in NSCLC. Expert Opin Drug Metab Toxicol.

[24] Park S.R., Kong S.Y., Nam B.H., Choi I.J., Kim C.G., Lee J.Y. (2011). *CYP2A6* and *ERCC1* polymorphisms correlate with efficacy of S-1 plus cisplatin in metastatic gastric cancer patients. Br J Cancer.

[25] Daigo S., Takahashi Y., Fujieda M., Ariyoshi N., Yamazaki H., Koizumi W. (2002). A novel mutant allele of the *CYP2A6* gene (*CYP2A6**11) found in a cancer patient who showed poor metabolic phenotype towards tegafur. Pharmacogenetics.

[26] Oscarson M., McLellan R.A., Gullstén H., Agúndez J.A., Benítez J., Rautio A. (1999). Identification and characterisation of novel polymorphisms in the *CYP2A6* locus: implications for nicotine metabolism. FEBS Lett.

[27] McGraw J., Waller D. (2012). Cytochrome P450 variations in different ethnic populations. Expert Opin Drug Metab Toxicol.

[28] Yang L., Yang Y., Qin Q., Zhou A., Zhao J., Wang J. (2014). Dose-finding study on adjuvant chemotherapy with S-1 plus oxaliplatin for gastric cancer. Mol Clin Oncol.

[29] Yang L., Yang Y., Qin Q., Zhou A., Zhao J., Wang J. (2015). Evaluation of the optimal dosage of S-1 in adjuvant SOX chemotherapy for gastric cancer. Oncol Lett.

[30] Yang L., Song Y., Zhou A.P., Qin Q., Chi Y., Huang J. (2013). A phase II trial of oxaliplatin plus S-1 as a first-line chemotherapy for patients with advanced gastric cancer. Chin Med J (Engl).

[31] Duffaud F., Therasse P. (2000). New guidelines to evaluate the response to treatment in solid tumors. Bull Cancer.

[32] Barrett J.C., Fry B., Maller J., Daly M.J. (2005). Haploview: analysis and visualization of LD and haplotype maps. Bioinformatics.

[33] Abecasis G.R., Cookson W.O. (2000). GOLD-graphical overview of linkage disequilibrium. Bioinformatics.

