# Supplementary material for: CYP2A6 Polymorphisms Associate with Outcomes of S-1 Plus Oxaliplatin Chemotherapy in Chinese Gastric Cancer Patients
Source: Genomics Proteomics Bioinformatics. 2017 Aug 12;15(4):255–62. doi: 10.1016/j.gpb.2016.11.004 (PMC5582793; doi:10.1016/j.gpb.2016.11.004)
Supplement: Supplementary Table S1 — Summary of CYP2A6 SNPs identified in the current study [file mmc1.rtf]

Table S1  Summary of CYP2A6 SNPs identified in the current study
SNP	Gene	dbSNP ID	Change	Allele	Functional annotation	MAF	P value	No. (%) of patients with SNP	
M01	CYP2A6	rs28399468	G>T	*8,*10,*37	R485L	0.062	0.012	6 (10.0)	
M02	CYP2A6	rs5031017	G>T	*5	G479V	0.038	0.077	4 (6.7)	
M03	CYP2A6	rs5031016	T>C	*7,*10,*19,*36,*37	I471T	0.038	0.077	4 (6.7)	
M04	CYP2A6	rs150586234	C>T	—	Synonymous	0.185	0.677	23 (38.3)	
M05	CYP2A6	rs771265125	C>T	—	Synonymous	0.146	0.334	19 (31.7)	
M06	CYP2A6	rs779290232	A>G	—	Synonymous	0.085	1.000	11 (18.3)	
M07	CYP2A6	rs762887319	T>C	—	M368T	0.015	1.000	2 (3.3)	
M08	CYP2A6	rs200267449	G>A	—	A347T	0.054	0.156	6 (10.0)	
M09	CYP2A6	rs58571639	C>T	—	R311C	0.177	1.000	21 (35.0)	
M10	CYP2A6	rs2644907	C>T	—	Synonymous	0.169	0.675	21 (35.0)	
M11	CYP2A6	rs60988093	G>A	—	Synonymous	0.154	1.000	19 (31.7)	
M12	CYP2A6	rs60823196	G>C	—	G301A	0.154	0.633	18 (30.0)	
M13	CYP2A6	rs4997557	C>G	—	T294S	0.185	0.030	19 (31.7)	
M14	CYP2A6	rs2644906	G>A	—	V292M	0.054	0.156	6 (10.0)	
M15	CYP2A6	rs2644905	A>G	—	Synonymous	0.154	1.000	19 (31.7)	
M16	CYP2A6	rs139639589	G>A	—	R274H	0.077	0.035	8 (13.3)	
M17	CYP2A6	rs55805386	T>C	—	Synonymous	0.054	0.114	6 (10.0)	
M18	CYP2A6	rs140471703	G>A	*41	R265Q	0.146	0.021	15 (25.0)	
M19	CYP2A6	rs138978736	C>A	—	Q239K	0.100	0.107	11 (18.3)	
M20	CYP2A6	rs111033610	T>C	*11	S223P	0.031	1.000	4 (6.7)	
M21	CYP2A6	rs199515342	C>T	*34	R64C	0.123	0.581	16 (26.7)	
M22	CYP2A6	rs200554095	A>T	*34	I61F	0.062	1.000	8 (13.3)	
Note: P values were generated based on Hardy-Weinberg equilibrium. MAF, minor allele frequency.
