# Supplementary material for: CYP2A6 Polymorphisms Associate with Outcomes of S-1 Plus Oxaliplatin Chemotherapy in Chinese Gastric Cancer Patients
Source: Genomics Proteomics Bioinformatics. 2017 Aug 12;15(4):255–62. doi: 10.1016/j.gpb.2016.11.004 (PMC5582793; doi:10.1016/j.gpb.2016.11.004)
Supplement: Supplementary Table S2 — Correlation between other toxicity phenotypes and CYP2A6 SNPs [file mmc2.docx]

**Table S2 Correlation between other toxicity phenotypes and *CYP2A6* SNPs**

| **SNP** | **dbSNP ID** | **Nausea** | | **Vomiting** | | **Thrombocytopenia** | | **Neutropenia** | |
| --- | --- | --- | --- | --- | --- | --- | --- | --- | --- |
|  |  | ***P* value** | **OR (CI 95%)** | ***P* value** | **OR (CI 95%)** | ***P* value** | **OR (CI 95%)** | ***P* value** | **OR (CI 95%)** |
| M01 | rs28399468 | 0.96 | 0.96 (0.22-4.19) | 0.72 | 0.63 (0.12-3.28) | 1 | 0.61 (0.07-5.25) | 0.67 | 0.41 (0.04-3.54) |
| M02 | rs5031017 | 1.07 | 1.07 (0.29-4.00) | 0.49 | 0.46 (0.09-2.29) | 0.68 | 0.47 (0.06-3.89) | 0.44 | 0.31 (0.03-2.62) |
| M03 | rs5031016 | 0.08 | nan | 0.09 | nan | 0.59 | nan | 1 | 0.60 (0.06-5,33) |
| M04 | rs150586234 | 0.80 | 1.20 (0.44-3.21) | 0.29 | 0.47 (0.15-1.52) | 0.75 | 1.21 (0.36-4.05) | 1 | 1.11 (0.36-3.36) |
| M05 | rs771265125 | 0.24 | 2.00 (0.63-6.36) | 0.76 | 1.25 (0.38-4.08) | 1 | 0.79 (0.16-3.80) | 0.73 | 1.41 (0.53-3.88) |
| M06 | rs779290232 | 1 | 1.17 (0.053-5.18) | 1 | 0.64 (0.06-6.37) | 0.15 | 4.73 (0.63-35.39) | 0.57 | nan |
| M07 | rs762887319 | 0.35 | 1.60 (0.64-3.96) | 0.33 | 1.65 (0.66-4.14) | 0.13 | 2.32 (0.82-6.48) | 0.59 | 1.43 (0.51-3.88) |
| M08 | rs200267449 | 0.81 | 1.13 (0.44-2.88) | 0.46 | 1.44 (0.56-3.70) | 0.24 | 1.86 (0.65-5.45) | 0.42 | 1.54 (0.56-4.22) |
| M09 | rs58571639 | 1 | 1.61 (0.10-26.37) | 0.11 | NA | 1 | nan | 0.43 | 3.12 (0.19-51.52) |
| M10 | rs2644907 | 0.71 | 0.63 (0.12-3.35) | 0.68 | 1.5 (0.32-7.02) | 1 | 0.72 (0.08-6.32) | 0.06 | 4.52 (0.95-21.42) |
| M11 | rs60988093 | 0.62 | 1.38 (1.53-3.60) | 0.60 | 1.37 (0.51-3.65) | 0.20 | 2.19 (0.74-6.46) | 0.57 | 1.38 (0.48-3.97) |
| M12 | rs60823196 | 0.71 | 0.63 (0.12-3.35) | 1 | 0.77 (0.14-4.14) | 1 | 0.72 (0.08-6.32) | 0.36 | 2.43 (0.51-11.5) |
| M13 | rs4997557 | 0.82 | 1.18 (0.48-2.90) | 0.81 | 1.21 (0.49-3.06) | 0.15 | 2.16 (0.78-5.99) | 0.79 | 0.77 (0.26-2.26) |
| M14 | rs2644906 | 0.62 | 1.38 (0.53-3.60) | 1 | 1.06 (0.39-2.89) | 0.52 | 1.60 (0.52-4.92) | 1 | 1.02 (0.34-3.08) |
| M15 | rs2644905 | 0.62 | 1.38 (0.53-3.60) | 0.60 | 1.37 (0.51-3.65) | 0.20 | 2.19 (0.74-6.46) | 0.57 | 1.38 (0.48-3.96) |
| M16 | rs139639589 | 1 | 1.07 (0.17-6.63) | 0.66 | 0.47 (0.05-4.40) | 0.22 | 3.12 (0.49-19.8) | 0.33 | nan |
| M17 | rs55805386 | 0.16 | nan | 0.16 | nan | 0.58 | nan | 0.33 | nan |
| M18 | rs140471703 | 0.82 | 1.18 (0.49-2.90) | 0.47 | 1.51 (0.61-3.75) | 0.38 | 1.63 (0.57-4.68) | 0.29 | 1.70 (0.65-4.47) |
| M19 | rs138978736 | 1 | 0.92 (0.34-2.53) | 0.79 | 1.16 (0.42-3.21) | 0.75 | 1.21 (0.36-4.05) | 1 | 1.11 (0.36-3.36) |
| M20 | rs111033610 | 0.20 | 0.33 (0.068-1.59) | 1 | 1.12 (0.31-4.08) | 0.11 | 2.83 (0.76-10.58) | 1 | 1.16 (0.28-4.67) |
| M21 | rs199515342 | 0.81 | 0.83 (0.32-2.12) | 0.62 | 1.32 (0.52-3.35) | 0.37 | 1.75 (0.60-5.04) | 0.10 | 2.34 (0.90-6.11) |
| M22 | rs200554095 | 0.71 | 0.51 (0.10-6.63) | 0.71 | 0.63 (0.12-3.28) | 0.63 | 1.51 (0.29-8.01) | 1 | 1.02 (0.19-5.33) |

*Note*: SNP, single nucleotide polymorphism; OR, odds ratio; CI, confidence interval; nan, not a number; na, not available. *P* values were generated by using 2-tailed Fisher’s exact test.
