# Supplementary material for: CYP2A6 Polymorphisms Associate with Outcomes of S-1 Plus Oxaliplatin Chemotherapy in Chinese Gastric Cancer Patients
Source: Genomics Proteomics Bioinformatics. 2017 Aug 12;15(4):255–62. doi: 10.1016/j.gpb.2016.11.004 (PMC5582793; doi:10.1016/j.gpb.2016.11.004)
Supplement: Supplementary Table S3 — List of CYP2A6 primers used in the current study [file mmc3.rtf]

Table S3  List of CYP2A6 primers used in the current study
Gene	Region	Forward primer (3'–5')	Reverse primer (3'–5')	
CYP2A6	Exon 1	CCCTCCTGAAGTACCACAGAT	TTCTCACAGTCAGGGAGCTG	
CYP2A6	Exon 2	TGGAGTTTTGGAGTTTCAGC	ATGGAGAGAGAGGGGAAAGA	
CYP2A6	Exon 3	TCTCTCTCTCTCTACCTCGACA	CCTTGTTGAGCCAAATTCC	
CYP2A6	Exon 4	AGAAGGAAAACACCCAGGAC	GGGAGCATCTGTTGAGCTAT	
CYP2A6	Exon 5	CCTGGTACCTAACCTTCCTGA	GACGGGGTTTGACCATGT	
CYP2A6	Exon 6	AAGGACAGATGGTCAGCAAG	CTGACCAATCAGTGCAGACA	
CYP2A6	Exon 7	GGTCATCCCCTAAGTTCCTC	AAGGGGGCTTCTGTTTCTTA	
CYP2A6	Exon 8	CCCAAACTTCCTGTTTCAGA	TGCCGTCATCTCCTTTTTAG	
CYP2A6	Exon 9	GAAAGAAATTGAGGCTCAGG	AGAAGGCTATGGGCACAGAT	
Note: The GenBank accession number for CYP2A6 sequence197100415.
